# Antiviral Toll-like Receptor Signaling in Non-Parenchymal Liver Cells Is Restricted to TLR3

**DOI:** 10.3390/v14020218

**Published:** 2022-01-24

**Authors:** Melanie Werner, Stefan Schefczyk, Martin Trippler, Juergen W. Treckmann, Hideo A. Baba, Guido Gerken, Joerg F. Schlaak, Ruth Broering

**Affiliations:** 1 Department of Gastroenterology, Hepatology and Transplant Medicine, University Hospital of Essen, University Duisburg-Essen, Hufelandstr. 55, 45147 Essen, Germany; melanie.werner@uni-due.de (M.W.); stefan.schefczyk@uni-due.de (S.S.); Martin.Trippler@uk-essen.de (M.T.); guido.gerken@helios-gesundheit.de (G.G.); joerg.schlaak@ob.ameos.de (J.F.S.); 2 Department of General-, Visceral- and Transplantation-Surgery, University Hospital of Essen, University of Duisburg-Essen, Hufelandstr. 55, 45147 Essen, Germany; juergen-walter.treckmann@uk-essen.de; 3 Institute of Pathology, University Hospital of Essen, University of Duisburg-Essen, Hufelandstr. 55, 45147 Essen, Germany; hideo.baba@uk-essen.de; 4 Helios Hospital, Gastroenterology, Hepatology and Palliative Medicine, Robert-Koch-Straße 2, 42549 Velbert, Germany; 5 AMEOS Hospital, St. Clemens, Internal Medicine—Hepatology, Gastroenterology, Infectiology and Diabetology, Wilhelmstr. 34, 46145 Oberhausen, Germany

**Keywords:** primary human non-parenchymal liver cells, hepatitis C virus, Toll-like receptors, interferons, Kupffer cells, liver sinusoidal endothelial cells, hepatic stellate cells

## Abstract

The role of non-parenchymal liver cells as part of the hepatic, innate immune system in the defense against hepatotropic viruses is not well understood. Here, primary human Kupffer cells, liver sinusoidal endothelial cells and hepatic stellate cells were isolated from liver tissue obtained after tumor resections or liver transplantations. Cells were stimulated with Toll-like receptor 1–9 ligands for 6–24 h. Non-parenchymal liver cells expressed and secreted inflammatory cytokines (IL6, TNF and IL10). Toll-like receptor- and cell type-specific downstream signals included the phosphorylation of NF-κB, AKT, JNK, p38 and ERK1/2. However, only supernatants of TLR3-activated Kupffer cells, liver sinusoidal endothelial cells and hepatic stellate cells contained type I and type III interferons and mediated an antiviral activity in the interferon-sensitive subgenomic hepatitis C virus replicon system. The antiviral effect could not be neutralized by antibodies against IFNA, IFNB nor IFNL, but could be abrogated using an interferon alpha receptor 2-specific neutralization. Interestingly, TLR3 responsiveness was enhanced in liver sinusoidal endothelial cells isolated from hepatitis C virus-positive donors, compared to uninfected controls. In conclusion, non-parenchymal liver cells are potent activators of the hepatic immune system by mediating inflammatory responses. Furthermore, liver sinusoidal endothelial cells were identified to be hyperresponsive to viral stimuli in chronic hepatitis C virus infection.

## 1. Introduction

The liver is one of the largest organs in the body, with diverse metabolic and immunologic functions. The liver cell population is constituted of ~60% parenchymal cells (hepatocytes) and ~40% non-parenchymal liver cells (NPC). The NPC fraction contains about 15% Kupffer cells (KC), 19% liver sinusoidal endothelial cells (LSEC), 5% hepatic star cells (HSC) and 1% lymphocytes and leukocytes, calculated for the entire cell population [1,2,3,4]. KCs represent the liver-resident macrophage population localized in the portal areas. These cells have a high phagocytic activity to remove pathogens like viruses, bacteria and endotoxins from the blood stream [5,6]. LSECs form a fenestrated lining within the hepatic sinusoids that represents a barrier between the sinusoidal lumen and the space of Disse [7,8], where HSCs are located [9]. The main function of HSC in a quiescent state is the storage of vitamins. After activation HSC start to synthesize large amounts of collagen and extracellular matrix proteins [10]. Furthermore, NPC have been described to play a crucial role for pathogen defense and immune tolerance induction [11]. As part of the innate immune system, NPC are equipped with a set of pattern recognition receptors, such as Toll-like receptors (TLR), which mediate innate immune responses after recognition of pathogen-associated molecular patterns, as shown for murine [12,13] and human cells [14].

In Broering et al., a functional TLR1-9 signaling has been observed in primary human hepatocytes (PHH). However, an antiviral response has been restricted to TLR3 stimulation. Furthermore, this paper indicates that chronic TLR3 activation leads to hyper- rather than hyposensitivity in hepatocytes [15]. We here question (I) whether antiviral TLR signaling in NPC is restricted too, (II) which antiviral mediators are induced and (III) if TLR3 hypersensitivity is observed in NPC isolated from HCV-infected patients.

## 2. Materials and Methods

### 2.1. Reagents

Toll-like receptor 1–9 ligands were obtained from InvivoGen (Toulouse, France), and used as previously described for PHH treatment [15]; TLR1/2 (palmitoyl-3-cysteine-serinelysine-4, Pam3CSK4, 4 µg/mL), TLR2 (heat-killed Listeria monocytogenes, HKLM, 10^8^ cells/mL), TLR3 (polyinosine:polycytidylic acid, poly(I:C), 50 µg/mL), TLR4 (Lipopolysaccharide, Escherichia coli 0111:B4 strain, LPS, 30 µg/mL), TLR5 (S. typhimurium flagellin, 2 µg/mL), TLR6/2 (Pam2CGDPKHPKSF, FSL-1, 1 µg/mL]), TLR7 (Gardiquimod, Gdq, 20 µg/mL), TLR8 (single-stranded RNA40, ssRNA40, 10 µg/mL) and TLR9 (CpG oligonucleotides, ODN2216, 31.8 µg/mL). Recombinant human IFNA A/D, IFNB1a and collagenase were purchased from Sigma (Heidenheim, Germany). Recombinant human IFNL2, IFNL3 and blocking antibodies against human IFNA and IFNB1 were obtained from eBioscience (Frankfurt, Germany). Neutralizing antibody against human interleukin 10 receptor beta (IL10RB) and cross-specific antibody that neutralize type III interferons were obtained from R&D systems (Wiesbaden, Germany). Blocking antibody against human interferon alpha/beta receptor 2 (IFNAR2) was obtained from PBL Assay Science (Piscataway, NJ, USA).

### 2.2. Isolation of Primary Human Liver Cells

Liver tissue was obtained after tumor resections or liver transplantations from HCV-infected patients (*n* = 10) or uninfected controls (*n* = 15). Patients’ characteristics are given in Supplementary Table S1. KC, LSEC and HSC were isolated and cultured as previously described [16]. Written informed consent was obtained from all patients, and the study was approved by the institutional review board (Ethics Committee) of the medical faculty at the University Duisburg-Essen.

### 2.3. Mouse Experiment

C57BL/6 mice were bred at the University Hospital of Essen, fed ad libidum and received humane care according to the criteria outlined in the Guide for the Care and Use of Laboratory Animals prepared by the National Academy of Sciences and published by the National Institutes of Health. The animal study design was approved by the local committee (*Landesamt für Natur, Umwelt und Verbraucherschutz*, LANUV). Poly(I:C) was administered intravenously at a dosage of 5 mg/kg body weight via tail vein injection, using 9-week-old male mice. Mice were scarified after 24 h and primary murine hepatocytes (PMH), LSEC, KC and remaining NPC (rNPC) were isolated as previously described [17]. Cells were directly lysed for analysis.

### 2.4. Cell Culture

Con1 cells [18] were cultured using Dulbecco’s modified Eagle’s medium supplemented with 10% fetal bovine serum (PAA, Pasching, Austria), 100 U/mL penicillin (PAA), 0.1 mg/mL streptomycin (PAA), 2 mM L-glutamine (PAA) and 300 U/mL G418 (Biochrome, Berlin, Germany).

### 2.5. RNA Isolation and One-Step Quantitative Reverse Transcription Polymerase Chain Reaction

Total RNA was extracted from cultured cells with QIAzol Lysis Reagent (Qiagen, Hilden, Germany) and the RNeasy Mini Kit (Qiagen) according to the manufacturer’s protocols. Specific primers for human *ACTB*, *MX1* and HCV used for one-step quantitative reverse transcription polymerase chain reaction (qRT-PCR) are given in Supplementary Table S2. All other genes were measured using QuantiTect Primer Assays (Qiagen). Calculated copy numbers were normalized to 100,000 copies of reference gene *ACTB* or *Gapdh* and given as mean ± standard deviation (SD).

### 2.6. Enzyme-Linked Immunosorbent Assay

Cell culture supernatants from NPC were collected 24 h after stimulation with TLR1–9 ligands and tested for secretion of IL6, TNF and IL10 by enzyme-linked immunosorbent assay (ELISA) according to the manufacturer’s instructions (R&D systems). Cell culture supernatants were further analyzed for secretion of IFNA (PBL Assay Science), IFNB1 (PBL Assay Science), IFNL1 (eBioscience) and IFNL2/3 (RayBiotech, Norcross, GA, USA).

### 2.7. LEGENDplex™

LEGENDplex™ (Biolegend, San Diego, CA, USA) is a bead-based immunoassay allowing us to quantify multiple soluble analytes simultaneously in biological samples using a flow cytometer. Here, whole-cell lysates from PMH, LSEC, KC and rNPC were prepared (500,000 cells per 100µ lysis buffer: 50 mM Tris/HCl (pH 7.4), 150 mM NaCl, 1 mM NaF, 1 mM EDTA, 0.1% SDS (*w*/*v*), 1% NP40 (*v*/*v*), 0.5% Na-Deoxycholat (*w*/*v*) and supplemented with phosphatase (Roche, Basel, Switzerland, CHE) and protease inhibitor (Roche, Basel, Switzerland)). LEGENDplex™ was performed according to the manufacturer´s instructions of (I) Anti-Virus response panel and (II) TH cytokine panel.

### 2.8. Western Blot

Western blot samples were prepared as previously described [15]. Blots were probed with specific antibodies (Supplementary Table S3) using 5% bovine serum albumin (PAA) in Tris-Buffered Saline and Tween 20. Chemiluminescence was induced using ECL Plus Western Blotting Detection Reagent (GE Healthcare, Piscataway, NJ, USA). Signals were detected using Fusion FX from Vilber.

### 2.9. Neutralization of Antiviral Activity of NPC

Cell culture supernatants of NPC treated with TLR1–9 ligands (1:2 dilution) for 24 h were co-cultured with con1 cells for 72 h. Total RNA was extracted and HCV replication was determined by qRT-PCR. Afterwards, con1 cells were treated with supernatants of polyinosine-polycytidylic acid (poly(I:C))-stimulated NPC in different dilutions (1:5; 1:10; 1:15) to determine the dilution with half maximal inhibitory effect. This dilution was used for neutralization experiments, supernatants of poly(I:C)-stimulated NPC or recombinant interferons were pre-incubated with neutralizing antibodies. In addition, con1 cells were pre-incubated with neutralizing antibodies against IFNAR2 or IL10RB. After pre-incubation for 3 h, con1 cells were stimulated with supernatants of NPC or interferons. Cells were cultured for 72 h and RNA was extracted. HCV replication was assessed by qRT-PCR.

### 2.10. Statistical Analysis

Data are shown as mean values ± SD. Differences between two groups were determined by unpaired Student’s *t*-test, including Welch’s correction if the F-test indicated unequal variances. *p*-values of <0.05 were considered to be statistically significant.

## 3. Results

### 3.1. NPC Express Toll-like Receptors

Gene expression of *TLR1–9* in unstimulated cells and cells stimulated with the respective ligand for 24 h was determined by qRT-PCR (Table 1). KC expressed high levels of *TLR1–4* and *TLR6–8* and moderate levels of *TLR5*. Furthermore, *TLR2* and *TLR3* were significantly induced after stimulation with HKLM and poly(I:C), respectively. In contrast, *TLR4–7* expression levels were significantly suppressed after stimulation with the respective ligands. LSEC and HSC expressed high levels of *TLR3* and *TLR4* and low levels of *TLR5–8*. After stimulation with the respective ligand, solely *TLR3* was significantly induced. Stimulation with the respective ligands led to suppression of *TLR4* and *TLR6* in LSEC or *TLR4*, *TLR5* and *TLR6* in HSC. In all three cell types, gene expression of *TLR9* was beneath the limit of detection.

### 3.2. NPC Express and Secrete Pro- and Anti-Inflammatory Cytokines in Response to TLR Ligand Stimulation

NPC and TLR play a pivotal role for the local innate immune system of the liver [4,19]. Since NPC showed TLR gene expression, we examined whether cultured NPC exhibit a functional TLR signaling. Therefore, primary human KC, LSEC and HSC were exposed to TLR ligands. After incubation for 6 h, RNA was extracted and gene expression of pro-inflammatory cytokines IL6 and TNF as well as anti-inflammatory cytokine IL10 was determined by qRT-PCR. Expression of IL6, TNF and IL10 was potently induced in response to TLR stimulation in a cell type-specific manner with maximum basal expression levels of TNF and IL10 in KC, whereas maximum IL6 expression was observed in HSC (Supplementary Table S4). In addition, cultured NPC were stimulated with TLR ligands for 24 h and supernatants were tested for IL6, TNF and IL10 secretion using ELISA (*n* = 3). In accordance with results from the gene expression analysis, KC seemed to be the most potent inducer of TNF and IL10, whereas HSC seemed to be the main source of pro-inflammatory cytokine IL6 (Table 2). In summary, TLR activation led to a cell type-specific induction of IL6, TNF and IL10 in human KC, LSEC and HSC demonstrating functional TLR signaling in NPC.

### 3.3. TLR Stimulation Activates Downstream Signaling in NPC

**Figure 1:**
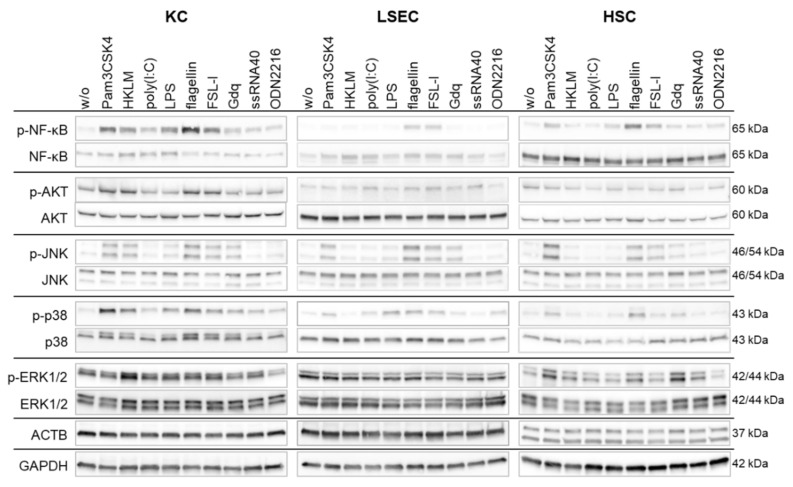
Cultured NPC trigger a functional downstream signaling after *TLR1–9* stimulation. Primary human KC, LSEC and HSC were stimulated with *TLR1–9* agonist for 30 min followed by extraction of total protein. Western blot analysis was performed with antibodies detecting ACTB or GAPDH as well as phosphorylated and total forms of NF κB, AKT, JNK, p38 and MAPK. Panels are representatives of three independent experiments. Original blots are given in the Supplementary Materials.

NPC express a number of pathogen recognition receptors to sense viral or bacterial structures. After recognition of these structures, activated cells initiate downstream signaling cascades to trigger defense mechanisms. Here, we analyzed activation (phosphorylation) of diverse signaling cascades after stimulation of TLR: nuclear factor kappa B (NF-κB), serine/threonine-protein kinase (AKT), c-Jun-amino-terminal kinases (JNK), protein38 (p38) and mitogen-activated protein kinase (MAPK). Therefore, cells were cultured and exposed to TLR ligands for 30 min. Whole cell lysates were collected and used for protein expression analysis by Western blot (*n* = 3). In KC, NF-κB and p38 signaling was activated by all TLR ligands. Furthermore, KC responded to Pam3CSK4-, FSL-1- and flagellin stimulation with activation of AKT and JNK (Figure 1). In LSEC, stimulation by flagellin and FSL-1 led to activation of NF-κB, JNK and p38 cascades. In addition, JNK and p38 were phosphorylated after stimulation with Pam3CSK4 and LPS, respectively (Figure 1). In HSC, activation by Pam3CSK4, flagellin and FSL-1 led to phosphorylation of NF-κB, JNK and p38 (Figure 1). In all tested LSEC and HSC the AKT pathway was continuously activated. Taken together, TLR stimulation led to a cell-type specific activation pattern of the TLR signaling cascade in NPC, thereby regulating inflammatory processes in the liver. Interestingly, poly(I:C) induced only weak or no NF-κB and MAPK activation, despite strong cytokine secretion given in Table 2.

### 3.4. Poly(I:C) Treatment of NPC Mediates an Antiviral Effect

**Figure 2:**
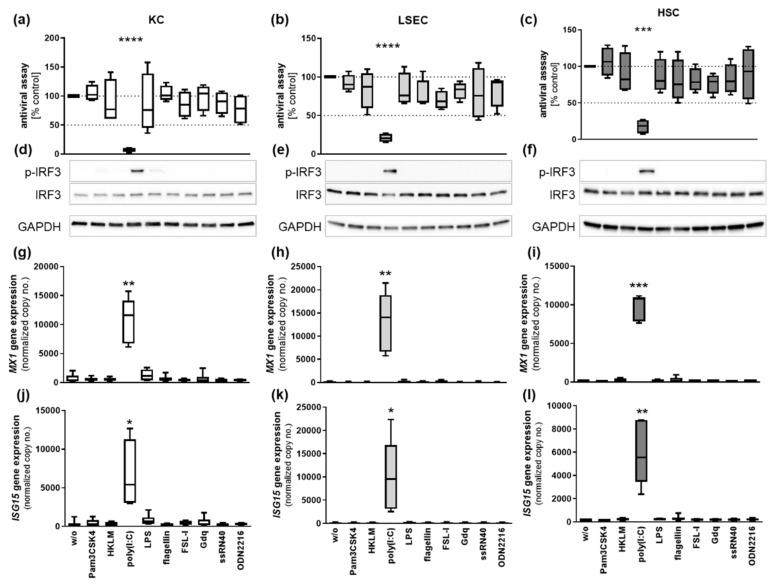
Poly(I:C)-stimulated NPC are potent suppressors of HCV replication in vitro. Primary (**a**) KC, (**b**) LSEC and (**c**) HSC were stimulated with *TLR1–9* agonists for 24 h and cell culture supernatants were collected (*n* = 4). Supernatants were co-cultured with the subgenomic HCV replicon system con1. After 72 h, RNA was extracted and HCV replication was determined by qRT-PCR. Cultured (**d**) KC, (**e**) LSEC and (**f**) HSC were stimulated with TLR1–9 agonists for 90 min, followed by extraction of total protein. Western blot analysis was performed with antibodies detecting GAPDH as well as phosphorylated and total IRF3. Original blots are given in the Supplementary Materials. (**g**,**j**) KC, (**h**,**k**) LSEC and (**i**,**l**) HSC were stimulated with TLR1–9 ligands for 6 h and RNA was extracted. Gene expression of *MX1* and *ISG15* was determined by qRT-PCR. Data represent copy numbers as mean ± SD normalized to 100,000 copies of reference gene *ACTB*. Asterisks indicate significant results (* *p*-value < 0.05, ** *p*-value < 0.01, *** *p*-value < 0.001, **** *p*-value < 0.0001).

We further examined the antiviral capacity of human NPC using the interferon-sensitive con1 cell line, which harbors a subgenomic HCV replicon. Therefore, NPC were stimulated with TLR1–9 ligands for 24 h and supernatants were collected. Con1 cells were co-cultured with supernatants of untreated or TLR ligand-treated NPC for 72 h. As control, con1 cells were directly stimulated either with 50 µg/mL poly(I:C) or recombinant IFNA A/D (Supplementary Figure S1). After incubation, RNA was extracted and HCV replication was assessed by qRT-PCR. Exclusively, supernatants of poly(I:C)-treated NPC triggered significant suppression of HCV replication (Figure 2). HCV replication was diminished about 95.3 ± 1.1%, 81.4 ± 11.3% and 74.7 ± 9.9% by poly(I:C)-treated KC, LSEC and HSC, respectively (Figure 2a–c). The replication level of HCV was not affected by direct poly(I:C) treatment (50 µg/mL), whereas exposure to recombinant IFNA A/D (10IU/mL) significantly revealed a strong suppression about 81.2%, underlining the IFN sensitivity of the used replicon system (Supplementary Figure S1).

### 3.5. Poly(I:C) Mediates IFN Responses in NPC

The transcription factor interferon regulatory factor 3 (IRF3) is a known regulator for poly(I:C)- and LPS-induced interferons [20,21] and transcriptional coactivator for inflammatory responses [22,23]. Here, NPCs were stimulated with TLR ligands for 90 min, and cell lysates were collected. The phosphorylation of IRF3 was determined by Western blot analysis. In KC, LSEC and HSC, IRF3 was strongly phosphorylated in response to poly(I:C) treatment (Figure 2d–f). Furthermore, mRNA expression of interferon-stimulated genes was analyzed in NPCs treated with TLR ligands for 6 h. KCs, LSECs and HSCs showed a low basal gene expression of MX1 and ISG15 (Figure 2g–l). Exposure to poly(I:C) led to a strong induction of both interferon-stimulated genes in KC (MX1 fold change (FC) 25.9 ± 3.6, *p*-value 0.0012; ISG15 FC 15.4 ± 3.8; *p*-value 0.0121), LSEC (MX1 FC 63.0 ± 12.1, *p*-value 0.0037; ISG15 FC 45.8 ± 13.4, *p*-value 0.0207) and HSC (MX1 FC 111.1 ± 19.1, *p*-value 0.0002; ISG15 FC 32.5 ± 6.0, *p*-value 0.0033). Interestingly, neither TLR4, nor TLR7–9 stimulation led to induction of interferon-stimulated genes. In addition to antiviral signaling, poly(I:C)-induced IRF3 activity might be responsible for cytokine production (Table 2) by enhancing NF-κB transcriptional activity.

**Figure 3:**
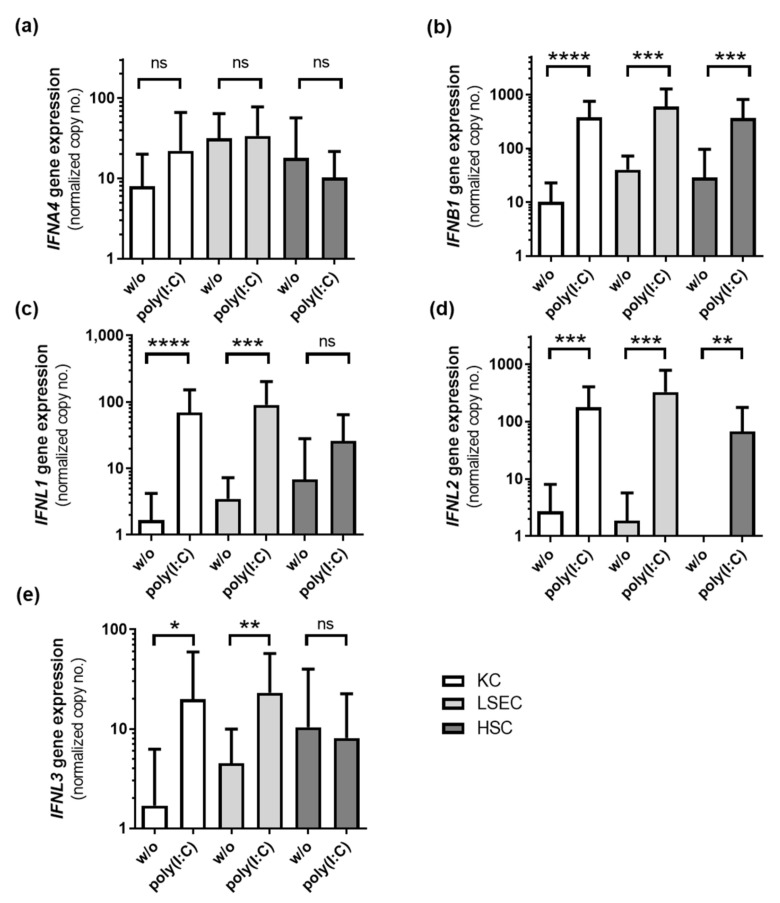
NPC express type I and type III interferons in response to poly(I:C) treatment. KCs, LSECs and HSCs isolated from liver resections and explants (*n* = 15) were stimulated with poly(I:C) for 6 h, and RNA was extracted. Gene expression of *IFNA4* (**a**), *IFNB1* (**b**), *IFNL1* (**c**), *IFNL2* (**d**) and *IFNL3* (**e**) was determined by RT qPCR. Data represent copy numbers as mean ± SD normalized to ACTB. Asterisks indicate significant results (* *p* < 0.05; ** *p* < 0.01; *** *p* < 0.001; **** *p*-value < 0.0001). Abbreviations: ns, not significant.

To determine the type of interferon, gene expression of different interferon genes was measured by qRT-PCR 6 h after poly(I:C) treatment (*n* = 15). The expression of IFNB1 was significantly induced in all NPC cell types and reached maximum expression values, compared to IFNL1 and IFNL2 expression levels (Figure 3). The secretion of IFNA, IFNB1, IFNL1 and IFNL2/3 was analyzed in poly(I:C)-treated NPC (*n* = 4) using ELISA. Therefore, cells were stimulated with poly(I:C) for 24 h followed by analysis of cell culture supernatants by ELISA. IFNA was not detectable in untreated cells and treated HSCs, but was slightly induced in poly(I:C)-stimulated KC and LSEC. IFNB1 was slightly produced in untreated cells and was significantly elevated after poly(I:C) treatment in KC, LSEC and HSC. Basal expression of IFNL1 and IFNL2/3 was below the limit of detection, but was significantly induced after poly(I:C) treatment in all three cell types (Table 3).

### 3.6. Poly(I:C)-Induced Antiviral Activity Is Mediated by Type I Interferons

**Figure 4:**
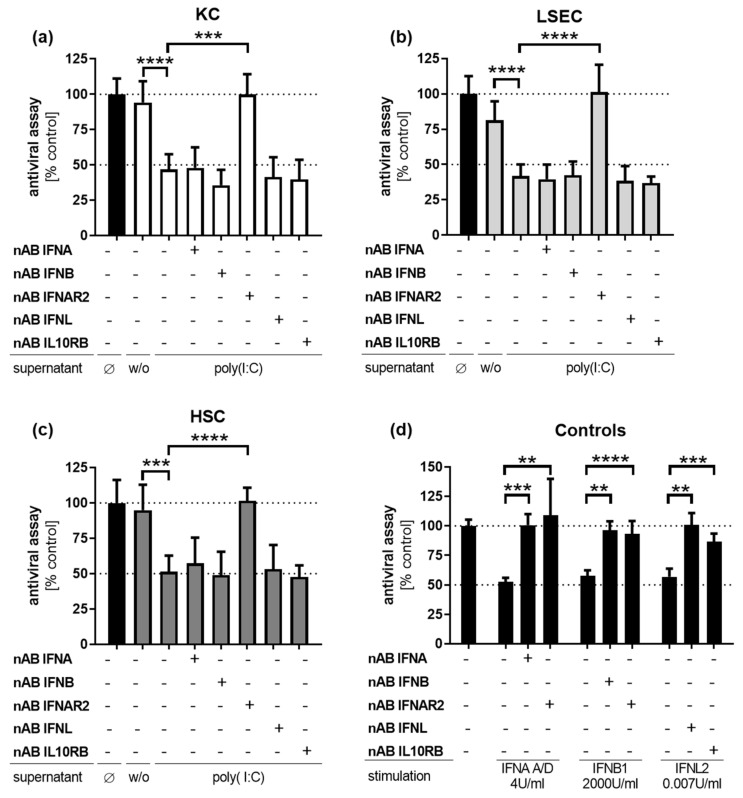
Antiviral effect of NPC is mediated by the type I interferon receptor. Cell culture supernatants from poly(I:C)-treated (**a**) KCs, (**b**) LSECs and (**c**) HSCs (*n* = 3) were preincubated with neutralizing antibodies against IFNA, IFNB1 or IFNL for 3 h. In parallel, con1 cells were preincubated with neutralizing antibodies against interferon receptors for 3 h. After incubation, con1 cells were exposed to preincubated, antibody-treated or untreated supernatants and cultured for 72 h. (**d**) Neutralizing antibodies were preincubated with respective interferons for 3 h and co-cultured with con1 cells for 72 h. Afterwards, RNA was extracted and HCV replication was determined by qRT-PCR (normalized to ACTB, mean ± SD). Asterisks indicate significant results (** *p* < 0.01; *** *p* < 0.001; **** *p*-value < 0.0001). Abbreviations: Ø no supernatants, w/o supernatants without treatment.

Since NPCs revealed strong antiviral response after poly(I:C) treatment, neutralizing experiments were performed to identify responsible mediators of the antiviral effect. For this purpose, supernatants of poly(I:C)-treated NPC were incubated with neutralizing antibodies specific for IFNA, IFNB1 or IFNL to neutralize interferon activity. In a second approach, both the IFNAR2 and the IFNL receptor chain IL10RB were neutralized in con1 cells to block type I or type III interferon signaling. For validation of antibody functionality, neutralizing antibodies were pre-incubated with the respective recombinant interferon and subsequently added to cultured con1 replicon cells. All three approaches were pre-incubated for 3 h. Afterwards, con1 cell culture media was replaced by the respective mixture. Cells were incubated for 72 h and RNA was extracted to analyze HCV replication by qRT-PCR. Treatment of con1 cells with diluted supernatants (inhibitory concentration of 50%, IC_50_) of poly(I:C)-stimulated KCs, LSECs and HSCs revealed a significant decrease of HCV replication (KC 53.2 ± 6.2%, LSEC 58.1 ± 4.7%; HSC 48.7 ± 6.6%) (Figure 4a–c). Interestingly, this suppressive effect was completely abrogated by blocking of IFNAR2 in all three cell types, while the antiviral effect was not affected by neutralization of IL10RB receptor chain. However, the antiviral effect of poly(I:C)-treated NPC was not impaired by blocking of IFNA, IFNB1 or IFNL. Hence, a synergistic effect of type I and type III interferons was analyzed by blocking combinations of interferons. Data analysis revealed the absence of any synergistic effects of combinations of type I and type III interferons (data not shown). The direct stimulation of con1 cells with interferons (IC_50_ dose) showed that HCV replication was potently diminished by stimulation with 2 U/mL IFNA A/D (47.6 ± 2.1%), 2000 U/mL IFNB1 (42.4 ± 2.7%) or 4.5 ng/mL IFNL2 (43.2 ± 4.0%) (Figure 4d). These antiviral effects could be neutralized using the specific antibodies, indicating the functionality of this assay.

### 3.7. LSEC from HCV-Positive Patients Are Hypersensitive to Poly(I:C) Treatment

**Figure 5:**
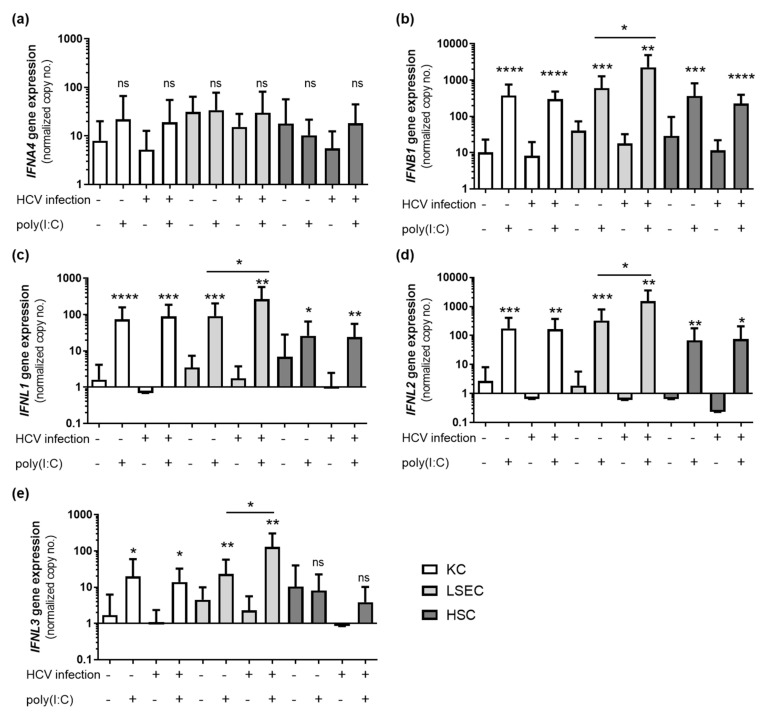
LSEC isolated from HCV-infected patients are more sensitive to poly(I:C). KCs, LSECs and HSCs isolated from uninfected controls (*n* = 15) or HCV-infected patients (*n* = 10) were stimulated with poly(I:C) for 6 h and RNA was extracted. Gene expression of interferons IFNA4 (**a**), IFNB1 (**b**), IFNL1 (**c**), IFNL2 (**d**) and IFNL3 (**e**) was determined by qRT-PCR. Data represent copy numbers as mean ± SD normalized to 100,000 copies of reference gene ACTB. Asterisks indicate significant results (* *p* < 0.05; ** *p* < 0.01; *** *p* < 0.001; **** *p* < 0.0001). Abbreviations: ns, not significant.

Interferon gene expression was analyzed in NPC isolated from HCV-infected patients (*n* = 10) or uninfected controls (*n* = 15). Therefore, cultured cells were stimulated with poly(I:C) for 6 h, RNA was extracted and used for detection of interferons by qRT-PCR. Patients’ characteristics are given as Supplementary Table S1. Comparison of interferon induction in poly(I:C)-stimulated cells from HCV-positive and -negative patients revealed, that LSECs, but not KCs and HSCs, obtained from HCV-positive donors were hypersensitive to poly(I:C) treatment. LSECs from HCV-positive patients expressed significantly higher levels of IFNB1 (Fold change (FC) 3.7, *p*-value 0.0139), IFNL1 (FC 3.4, *p*-value 0.0236), IFNL2 (FC 5.4, *p*-value 0.0203) and IFNL3 (FC 6.9, *p*-value 0.0157) (Figure 5). KCs and HSCs isolated from HCV-infected patients or uninfected controls showed no significant differences in response to Poly(I:C) treatment.

### 3.8. Poly(I:C) Response In Vivo Is Cell Type-Specific

**Figure 6:**
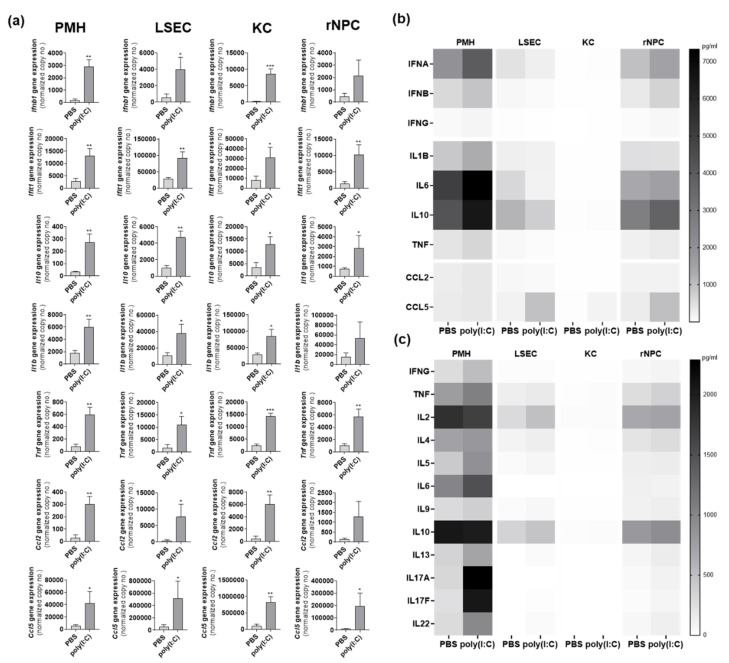
Poly(I:C)-induces a cell type-specific immune induction in vivo. PMH, LSEC, KC and rNPC were isolated from PBS- and poly(I:C)-treated 9-week-old C57BL/6 mice (group size *n* = 3). Gene expression of *Ifnb1, Ifit1, Il10, Il1b, Tnf, Ccl2* and *Ccl5* was determined by qRT-PCR (**a**). Data represent copy numbers as mean ± SD normalized to 100,000 copies of reference gene *Gapdh*. LEGENDplex^TM^ Anti-Virus response panel (**b**) and LEGENDplex^TM^ TH cytokine panel (**c**) was performed to determine protein levels (pg/mL) in cell lysates from PMH, LSEC, KC and rNPC from PBS- and poly(I:C)-treated mice (group size *n* = 3). Data given as heat maps of mean values (*n* = 3). Asterisks indicate significant results (* *p* < 0.05; ** *p* < 0.01; *** *p* < 0.001. Abbreviations: PBS, phosphate-buffered saline; PMH, primary mouse hepatocytes; rNPC, remaining non-parenchymal cells.

To investigate cell type-specific responses in vivo, 9-week-old C57BL/6 mice (*n* = 3) intravenously received poly(I:C). After 24 h parenchymal and non-parenchymal liver cells were prepared and 500,000 cells of PMH, LSEC, KC and the remaining NPC fraction, mainly containing HSC and leucocytes, were directly lysed for qRT-PCR or LEGENDplex^TM^ analysis. All four fractions showed poly(I:C)-induced gene expression (*Ifnb1, Ifit1, Il10, Il1b, Tnf, Ccl2* and *Ccl5*). In rNPC gene induction of *Ifnb1, Il1b* and *Ccl2* were not significant. Comparison of magnitudes of gene induction identified KCs to reach maximum gene expression for almost all measured genes (Figure 6a), except Ifit1 and Ccl2, which tended to have higher expression in LSEC. The LEGENDplex^TM^ data for the Anti-Virus response panel (Figure 6b) and TH cytokine panel (Figure 6c) are given as heat maps of mean values (*n* = 3). Herein, PMH showed the maximum protein abundance for poly(I:C)-induced IFNA, IFNB, IL1B, IL6, IL10, TNF, IL17A and IL17F. However, it might be suggested that LSEC and KC already secreted these cytokines within the 24 h after poly(I:C) exposure; so that would explain why intracellular cytokine levels at this time point were very low in LSEC and KC.

## 4. Discussion

The role of NPC as part of the innate immune system in the defense against hepatotropic viruses such as HCV is not well understood. Therefore, the aim of this study was to characterize the TLR signaling and antiviral capacity of primary human NPC. (I) Stimulation of TLR1–9 in NPC elicited secretion of inflammatory cytokines and activation of downstream signaling pathways as NF-κB, JNK and p38. However, antiviral potential of NPC was restricted to TLR3 activation that led to secretion of type I and type III interferons. (II) Although IFNAR2 was shown to mediate the antiviral effect, IFNA and IFNB1 did not seemed to be involved. (III) LSECs, but not KCs and HSCs, isolated from HCV-infected patients showed hyperresponsiveness to poly(I:C) treatment, as known for PHH [15].

NPC exert a functional role in the regulation of issues like liver fibrosis [24,25,26], cirrhosis or hepatocellular carcinoma during viral infections. Previous studies addressed TLR pathways in murine non-parenchymal cells and human hepatocytes [15,27]. The present study characterized the TLR signaling of human KCs, LSECs, and HSCs and their antiviral capacity, using an HCV-based model. It has been shown that primary murine KCs express *TLR1–9* (except TLR5), LSECs express *TLR1–8* (except TLR5) [28] and HSCs express *TLR1–9* [12] on the transcriptional level. There are limited data available describing the TLR system in human NPCs. Faure-Dupuy et al. have assessed the expression of *TLR1–9* in human parenchymal and non-parenchymal liver cells, however, microfluid high-throughput quantitative RT-PCR failed to visualize most of the TLRs in untreated cells. Western blots indicate that *TLR2* is only expressed on KCs, TLR3 and -4 are present in all cell types, TLR5 is expressed on HSCs and PHH, *TLR7* and -8 could be detected in KCs and LSECs and *TLR1* and -9 remain undetectable [14]. Another study has demonstrated through flow cytometry that human KCs express *TLR2*, *TLR3* and *TLR5* [29,30]. Herein, quantitative RT-PCR assays detected a verifiable *TLR1–8* gene expression in KCs; *TLR1–4* and *TLR6* expression in LSECs and HSCs (Table 1). Despite the low expression, stimulation with respective ligands led to phosphorylation of NF-κB, IRF3, AKT, JNK, p38 and ERK1/2, as well as the secretion of cytokines, suggesting a broader and cell type-specific TLR responsiveness, emphasizing KCs to be the most powerful TLR responder. Our study further indicated that the expression of *TLR3* was induced after treatment by its ligand poly(I:C), whereas expression of *TLR4*, *TLR5* and *TLR6* was suppressed after stimulation with LPS, flagellin and FSL-1, respectively. Continuous activation of the immune system is known to lead to hepatocyte injury and thus loss of liver function [11,31]. Therefore, suppression of TLR expression may play an important role for tolerance mechanisms of the liver.

In the present work, stimulation of TLR mediated activation of the pathway-related kinases JNK as well as p38 and especially the transcription factor NF-κB in primary human KCs, LSECs and HSCs. Activation of these pathways potently initiated expression of inflammatory cytokines on protein and transcriptional level. Previously, it has been shown that human KCs secrete proinflammatory cytokines such as IL6 or TNF and anti-inflammatory cytokine IL10 in response to *TLR4* stimulation by LPS [32,33]. Furthermore, it has been shown that murine KCs secreted TNF and IL6 after *TLR1–9* stimulation [28]. Murine LSEC secreted TNF in response to *TLR1–4*, *TLR6* and *TLR8–9* stimulation and IL6 after *TLR3–4* [28]. Murine HSC secreted TNF after TLR3-4 stimulation [12]. Comparison between data from primary human hepatocytes [15] and data from the present study show that KCs had the highest capacity to express TNF and IL10. However, HSCs showed the highest potential to express IL6. Thus, these data are in accordance with previous publications [33,34] that show secretion of IL10 and TNF by human or rat KCs, respectively. Production of the anti-inflammatory cytokine IL10 by KCs may also play a supporting role for induction of immune tolerance of the liver [35].

Type I IFN comprise the members IFNA, IFNB1, IFN-omega (IFNW1), IFN-epsilon (IFNE) and IFN-kappa (IFNK) in humans [36]. These types of interferons share the same receptor composed of two subunits, IFNAR1 and IFNAR2, to mediate downstream signaling [37]. IFNL, consist of the members IFNL1, IFNL2 and IFNL3 [38] as well as a more recently described member, termed IFNL4 [39]. All IFNL act through a receptor complex composed of two subunits interferon lambda receptor 1 and IL10RB [38,40]. IFNL1, IFNL2 and IFNL3 mediate an antiviral activity against HCV in vitro and in vivo [41,42,43]. Although, IFNL4 has been shown to induce expression of ISGs, however, it is strongly associated with the failure to clear HCV infection mediated by a so far unknown mechanism [39,44]. Previous data indicate that TLR3- and TLR4-activated murine KC and TLR3-activated murine LSEC trigger a strong HCV-suppressive effect by production of IFNB1 [27,28]. Type III interferons are capable of inhibiting HBV replication in a murine hepatocyte cell line (HBV-Met) and HCV replication in the human hepatocyte cell line Huh7 [41]. It has been shown that gene expression of type III, and not of type I interferons, is upregulated in liver biopsies of HCV-infected chimpanzees [45]. Furthermore, type III IFN gene expression is induced by stimulation with poly(I:C) [15,45]. Not much is known about the antiviral capacity of human NPC. Here, IFNA, IFNB, IFNL1, IFNL2 and IFNL3 were analyzed as potential mediators of an antiviral effect of NPCs, which exerted a strong TLR3-dependent antiviral potential that was mediated by type I interferon receptors, but not by IFNA or IFNB1 alone. However, the IFNA family consists of 12 subtypes, which all bind the same receptor, but significantly differ in their biological activities [46,47]. It might be possible that the IFNA-neutralizing antibody that was used in the present study is less effective against diverse IFNA subtypes, although neutralizing the recombinant IFNA A/D. Expression levels of other type I interferons as *IFNW1* and *IFNE* were low in NPCs, suggesting a minor role for the antiviral effect in NPCs (Supplementary Figure S2b). Gene expression of *IFNK* was not detectable in human NPCs (data not shown). In contrast to studies in primary human hepatocytes [15], NPCs did not show a predominant role of type III interferons [15]. The present study focused on the TLR system in human NPCs, however it is still an open question, whether other groups of virus-sensing pattern recognition receptors as RIG-like receptors and cytosolic DNA sensors induce antiviral responses in these cells.

In patients infected with HCV hepatic IFNL-induced ISG signatures are associated with chronic progression and non-response to IFN-based therapies [39], our previous work highlighted the role of hepatocytes in this context [15]. A hyperresponsiveness to poly(I:C) in PHH derived from HCV patients has been mimicked by repetitive poly(I:C) stimulation of uninfected PHH in vitro. Herein, IFN response increases upon repetitive stimulation, whereas the cytokine expression indicates tolerance induction [15]. The present study aimed to investigate hyperresponsivness in NPCs of HCV-infected patients. Only LSEC isolated from HCV-positive donors showed higher responsiveness to poly(I:C) treatment and expressed increased levels of interferons compared to cells isolated from uninfected controls. Suggesting that hypersensitivity might be caused by a permanent activation of TLR3 that increases interferon production in PHH and LSEC. The role of LSECs in unfavorable, hepatic ISG signature in chronic HCV infection needs to be further investigated, as (I) the antiviral mediator was type III interferon-independent and (II) an NPC-related ISG signature, is a favorable predictor in HCV infection [48].

The present study is focused on NPC isolation, based on primary material from diseased patients. Therefore, advantages of the state of the art NPC culture is accompanied by limitations. Liver tissue was obtained after tumor resection of patients under diverse treatments. Furthermore, transplant-derived tissue represents end-stage liver diseases including different grades of inflammation as well as diverse stages of cirrhosis. Hepatic preactivation of inflammatory processes likely includes mechanisms that resolve inflammation and promote tissue regeneration [49]. Differences in TLR-mediated responses of KC, LSEC or HSC might be attributable not only to HCV infection, but also to the deranged homeostatic inflammatory processes, associated with the stage of liver damage [49]. NPCs, especially KCs, isolated from diseased liver tissue might have lost their liver-specific tolerogenic phenotype [50], thereby promoting inflammatory responses in vitro. The present study lacks information on phenotypic markers, that indicate a natural or diseased state of the NPC.

## 5. Conclusions

Human NPCs preliminarily responded to TLR ligands by production of inflammatory cytokines. A TLR-induced antiviral effect in NPCs, however, was restricted to poly(I:C) treatment and seems to be mediated by type I IFNs. In accordance with data recently obtained from primary human hepatocytes, TLR3-mediated expression of IFNB, IFNL1, IFNL2 and IFNL3 was elevated in LSEC obtained from HCV-infected patients, compared to uninfected controls. These findings shed new light on the relevance of NPCs in the pathogenesis of HCV.

## Figures and Tables

**Table 1:** Primary human NPC express TLR.

KC (*n* = 6)	w/o	TLR stimulation	
Gene	Mean ± SD	Mean ± SD	*p*-Value
*TLR1*	819.3 ± 380.9	828.1 ± 552.8	0.974
*TLR2*	780.2 ± 347.8	4775.2 ± 3125.1	0.010
*TLR3*	651.2 ± 162.6	4759.3 ± 2296.9	0.001
*TLR4*	678.7 ± 341.0	189.6 ± 53.6	0.005
*TLR5*	78.7 ± 51.4	9.1 ± 5.1	0.007
*TLR6*	481.8 ± 181.0	232.0 ± 120.8	0.011
*TLR7*	220.1 ± 146.5	48.2 ± 23.5	0.015
*TLR8*	540.3 ± 417.9	193.4 ± 95.8	0.071
*TLR9*	nd	nd	
LSEC (*n* = 6)	w/o	TLR stimulation	
Gene	Mean ± SD	Mean ± SD	*p*-Value
*TLR1*	57.9 ± 36.5	48.5 ± 32,3	0.639
*TLR2*	94.0 ± 50.5	159.1 ± 123,0	0.254
*TLR3*	386.4 ± 131.5	4013.5 ± 1413.6	<0.001
*TLR4*	899.5 ± 555,5	195.2 ± 30.4	0.010
*TLR5*	6.6 ± 3.2	5.0 ± 2.2	0.304
*TLR6*	127.7 ± 47.0	78.9 ± 27.7	0.043
*TLR7*	6.1 ± 3.7	3.5 ± 2.9	0.199
*TLR8*	7.8 ± 5.1	7.1 ± 5.1	0.816
*TLR9*	nd	nd	
HSC (*n* = 6)	w/o	TLR stimulation	
Gene	Mean ± SD	Mean ± SD	*p*-Value
*TLR1*	81.6 ± 48.7	48.4 ± 11.0	0.129
*TLR2*	115.8 ± 156.8	87.7 ± 72.5	0.699
*TLR3*	629.8 ± 224.4	4280.3 ± 1079.0	<0.001
*TLR4*	328.0 ± 136.4	146.9 ± 57.8	0.009
*TLR5*	4.7 ± 3.43	1.5 ± 0.7	0.040
*TLR6*	114.5 ± 28.2	72.5 ± 17.9	0.006
*TLR7*	9.0 ± 8.1	6.0 ± 4.9	0.439
*TLR8*	6.2 ± 7.6	1.4 ± 0.7	0.153
*TLR9*	nd	nd	

Gene expression of *TLR1–9* was determined in untreated NPC or in cells treated with the respective TLR ligand for 24 h (*n* = 6). Data represent copy numbers as mean ± SD normalized to 100,000 copies of reference gene ACTB. Abbreviations: HSC, hepatic stellate cells; KC, Kupffer cells; LSEC, liver sinusoidal endothelial cells; nd, not detectable; SD, standard deviation; TLR, Toll-like receptor; w/o (without treatment).

**Table 2:** NPC secrete inflammatory cytokines in response to TLR stimulation.

KC (*n* = 3)	IL6		TNF		IL10	
Stimulation	pg/mL	*p*-Value	pg/mL	*p*-Value	pg/mL	*p*-Value
w/o	1647.4 ± 1095.5		10.2 ± 7.4		54.3 ± 12.3	
Pam3CSK4	4729.3 ± 3837.2	0.231	522.8 ± 371.9	0.063	145.4 ± 47.8	0.020
HKLM	17,167.7 ± 5.829.6	0.005	664.4 ± 339.7	0.021	377.0 ± 192.1	0.033
poly(I:C)	12,772.3 ± 6479.3	0.030	96.0 ± 42.1	0.016	47.5 ± 9.4	0.459
LPS	15,717.3 ± 9325.7	0.047	706.3 ± 349.9	0.018	189.9 ± 50.9	0.005
flagellin	27,573.0 ± 12,656.3	0.016	957.0 ± 309.9	0.003	365.3 ± 145.5	0.014
FSL-I	9553.0 ± 3537.5	0.010	500.3 ± 379.5	0.076.	131.2 ± 27.2	0.003
Gdq	7703.3 ± 6502.5	0.169	652.1 ± 271.9	0.009	293.8 ± 161.1	0.050
ssRNA40	4926.4 ± 1678.9	0.020	486.7 ± 367.5	0.075	133.1 ± 43.6	0.025
ODN2216	1506.5 ± 684.2	0.855	54.5 ± 20.1	0.011	59.2 ± 10.6	0.617
LSEC (*n* = 3)	IL6		TNF		IL10	
Stimulation	pg/mL	*p*-Value	pg/mL	*p*-Value	pg/mL	*p*-Value
w/o	223.5 ± 44.2		10.2 ± 7.4		6.0 ± 0.7	
Pam3CSK4	280.1 ± 104.6	0.418	522.8 ± 371.9	0.063	9.8 ± 0.5	<0.001
HKLM	575.4 ± 323.5	0.119	664.4 ± 339.7	0.021	11.6 ± 1.6	0.001
poly(I:C)	3582.7 ± 81.8	<0.001	96.0 ± 42.1	0.016	9.1 ± 2.4	0.083
LPS	2310.8 ± 399.2	<0.001	706.3 ± 349.9	0.018	17.1 ± 1.7	<0.001
flagellin	2417.7 ± 740.5	0.004	957.0 ± 309.9	0.003	26.7 ± 4.5	<0.001
FSL-I	2408.8 ± 821.7	0.006	500.3 ± 379.5	0.076	16.0 ± 1.9	<0.001
Gdq	557.8 ± 186.0	0.025	652.1 ± 271.9	0.009	9.2 ± 1.2	0.004
ssRNA40	1578.1 ± 1132.4	0.093	486.7 ± 367.5	0.075	9.2 ± 2.8	0.110
ODN2216	195.4 ± 28.9	0.381	54.5 ± 20.1	0.011	6.5 ± 1.4	0.605
HSC (*n* = 3)	IL6		TNF		IL10	
Stimulation	pg/mL	*p*-Value	pg/mL	*p*-Value	pg/mL	*p*-Value
w/o	4402.6 ± 1393.1		17.5 ± 0.2		11→0.3	
Pam3CSK4	13,136.6 ± 3321.4	0.004	18.1 ± 0.5	0.159	1.4 ± 0.3	0.335
HKLM	14,080.1 ± 4014.4	0.007	18.8 ± 1.0	0.091	1.7 ± 0.3	0.119
poly(I:C)	66,950.4 ± 66,005.2	<0.001	20.5 ± 2.9	0.146	1.1 ± 0.2	0.845
LPS	54,034.3 ± 12,470.9	0.001	18.4 ± 0.5	0.029	1.9 ± 0.5	0.092
flagellin	55,203.0 ± 11,846.5	0.001	20.7 ± 0.3	<0.001	2.5 ± 0.5	0.003
FSL-I	15,057.0 ± 4792.6	0.011	18.4 ± 0.3	0.009	1.9 ± 0.7	0.119
Gdq	7390.0 ± 2607.3	0.120	19.2 ± 1.2	0.070	1.7 ± 0.5	0.153
ssRNA40	5127.7 ± 1352.6	0.532	17.7 ± 0.3	0.318	1.5 ± 0.5	0.296
ODN2216	5575.7 ± 1158.2	0.289	18.4 ± 0.3	0.003	1.7 ± 0.7	0.217

Secretion of inflammatory cytokines was determined in untreated NPC (w/o) or in cells treated with TLR ligands for 24 h. Data represent concentrations in pg/mL as mean ± SD. Abbreviations: FSL-I; synthetic lipoprotein (*TLR2/6* ligand); Gdq, Gardiquimod (*TLR7* ligand); HKLM, Heat Killed Listeria monocytogenes (*TLR2* ligand); HSC, hepatic stellate cells; KC, Kupffer cells; LPS, lipopolysaccharide (*TLR4* ligand); LSEC, liver sinusoidal endothelial cells; ODN2216, synthetic single-stranded DNA (TLR9 ligand); Pam3CSK4, synthetic triacylated lipopeptide (*TLR1*/*2* ligand); poly(I:C), polyinosinic-polycytidylic acid (*TLR3* ligand); SD, standard deviation; ssRNA40, single-stranded RNA oligonucleotide (*TLR8* ligand); TLR, Toll-like receptor; w/o (without treatment).

**Table 3:** NPC secrete type I and type III interferons in response to poly(I:C) treatment.

	KC			LSEC			HSC		
	w/o	Poly(I:C)	*p*-Value	w/o	Poly(I:C)	*p*-Value	w/o	Poly(I:C)	*p*-Value
IFNA [pg/mL]	nd	24.1 ± 8.1	-	nd	8.1 ± 2.5	-	nd	nd	-
IFNB1 [pg/mL]	3.7 ± 1.0	22.2 ± 15	0.042	5.1 ± 0.8	133.1 ± 90.8	0.026	4.6 ± 1.0	60.0 ± 45.0	0.043
IFNL1 [pg/mL]	0.9 ± 1.2	99.3 ± 67.2	0.022	0.8 ± 1.0	98.2 ± 75.4	0.036	nd	54.4 ± 21.2	-
IFNL2/3 [pg/mL]	nd	32.8 ± 9.1	-	2.7 ± 1.7	28.8 ± 18.6	-	nd	288.6 ± 293.6	-

Secretion of type I and type III interferons was determined in untreated NPC (w/o) or in cells treated with poly(I:C) for 24 h (*n* = 4). Data represent concentrations in pg/mL as mean ± SD. Abbreviations: HSC, hepatic stellate cells; IFN, interferon; KC, Kupffer cells; LSEC, liver sinusoidal endothelial cells, nd, not detectable; poly(I:C), polyinosinic-polycytidylic acid (*TLR3* ligand).

## Data Availability

Not Applicable.

## Supplementary Materials

The following are available online at https://www.mdpi.com/article/10.3390/v14020218/s1, Figure S1: HCV replicon system con1 does not respond to poly(I:C), Figure S2: NPC show low expression of IFNW and IFNE, Table S1: Patients characteristics, Table S2: Primer sequences, Table S3: Primary and secondary antibodies, Table S4: TLR-induced gene expression of inflammatory cytokines, Original Western blots (Figure 1 and Figure 2).

## References

[1] Wisse E., van’t Noordende J.M., van der Meulen J., Daems W.T. (1976). The pit cell: Description of a new type of cell occurring in rat liver sinusoids and peripheral blood. Cell Tissue Res..

[2] Blouin A., Bolender R.P., Weibel E.R. (1977). Distribution of organelles and membranes between hepatocytes and nonhepatocytes in the rat liver parenchyma. A stereological study. J. Cell Biol..

[3] Kmiec Z. (2001). Cooperation of liver cells in health and disease. Adv. Anat. Embryol. Cell Biol..

[4] Racanelli V., Rehermann B. (2006). The liver as an immunological organ. Hepatology.

[5] Smedsrod B., De Bleser P.J., Braet F., Lovisetti P., Vanderkerken K., Wisse E., Geerts A. (1994). Cell biology of liver endothelial and Kupffer cells. Gut.

[6] Kolios G., Valatas V., Kouroumalis E. (2006). Role of Kupffer cells in the pathogenesis of liver disease. World J. Gastroenterol. WJG.

[7] Wisse E. (1970). An electron microscopic study of the fenestrated endothelial lining of rat liver sinusoids. J. Ultrastruct. Res..

[8] Wisse E., De Zanger R.B., Charels K., Van Der Smissen P., McCuskey R.S. (1985). The liver sieve: Considerations concerning the structure and function of endothelial fenestrae, the sinusoidal wall and the space of Disse. Hepatology.

[9] Friedman S.L. (2008). Hepatic stellate cells: Protean, multifunctional, and enigmatic cells of the liver. Physiol. Rev..

[10] Geerts A. (2001). History, heterogeneity, developmental biology, and functions of quiescent hepatic stellate cells. Semin. Liver Dis..

[11] Tiegs G., Lohse A.W. (2010). Immune tolerance: What is unique about the liver. J. Autoimmun..

[12] Wang B., Trippler M., Pei R., Lu M., Broering R., Gerken G., Schlaak J.F. (2009). Toll-like receptor activated human and murine hepatic stellate cells are potent regulators of hepatitis C virus replication. J. Hepatol..

[13] Wu J., Meng Z., Jiang M., Pei R., Trippler M., Broering R., Bucchi A., Sowa J.-P., Dittmer U., Yang D. (2009). HBV suppresses toll-like receptor mediated innate immune responses in murine parenchymal and non-parenchymal liver cells. Hepatology.

[14] Faure-Dupuy S., Vegna S., Aillot L., Dimier L., Esser K., Broxtermann M., Bonnin M., Bendriss-Vermare N., Rivoire M., Passot G. (2018). Characterization of Pattern Recognition Receptor Expression and Functionality in Liver Primary Cells and Derived Cell Lines. J. Innate Immun..

[15] Broering R., Lutterbeck M., Trippler M., Kleinehr K., Poggenpohl L., Paul A., Gerken G., Schlaak J.F. (2014). Long-term stimulation of Toll-like receptor 3 in primary human hepatocytes leads to sensitization for antiviral responses induced by poly I:C treatment. J. Viral Hepat..

[16] Werner M., Driftmann S., Kleinehr K., Kaiser G.M., Mathe Z., Treckmann J.W., Paul A., Skibbe K., Timm J., Canbay A. (2015). All-In-One: Advanced preparation of Human Parenchymal and Non-Parenchymal Liver Cells. PLoS ONE.

[17] Liu J., Huang X., Werner M., Broering R., Yang D., Lu M. (2017). Advanced Method for Isolation of Mouse Hepatocytes, Liver Sinusoidal Endothelial Cells, and Kupffer Cells. Methods Mol. Biol..

[18] Lohmann V., Korner F., Koch J., Herian U., Theilmann L., Bartenschlager R. (1999). Replication of subgenomic hepatitis C virus RNAs in a hepatoma cell line. Science.

[19] Takeda K., Akira S. (2005). Toll-like receptors in innate immunity. Int. Immunol..

[20] Sato M., Tanaka N., Hata N., Oda E., Taniguchi T. (1998). Involvement of the IRF family transcription factor IRF-3 in virus-induced activation of the IFN-beta gene. FEBS Lett..

[21] Yoneyama M., Suhara W., Fujita T. (2002). Control of IRF-3 activation by phosphorylation. J. Interferon Cytokine Res. Off. J. Int. Soc. Interferon Cytokine Res..

[22] Kumari M., Wang X., Lantier L., Lyubetskaya A., Eguchi J., Kang S., Tenen D., Roh H.C., Kong X., Kazak L. (2016). IRF3 promotes adipose inflammation and insulin resistance and represses browning. J. Clin. Investig..

[23] Leung T.H., Hoffmann A., Baltimore D. (2004). One nucleotide in a kappaB site can determine cofactor specificity for NF-kappaB dimers. Cell.

[24] Meyer D.H., Bachem M.G., Gressner A.M. (1990). Modulation of hepatic lipocyte proteoglycan synthesis and proliferation by Kupffer cell-derived transforming growth factors type beta 1 and type alpha. Biochem. Biophys. Res. Commun..

[25] DeLeve L.D. (2014). Liver sinusoidal endothelial cells in hepatic fibrosis. Hepatology.

[26] Moreira R.K. (2007). Hepatic stellate cells and liver fibrosis. Arch. Pathol. Lab. Med..

[27] Broering R., Wu J., Meng Z., Hilgard P., Lu M., Trippler M., Szczeponek A., Gerken G., Schlaak J.F. (2008). Toll-like receptor-stimulated non-parenchymal liver cells can regulate hepatitis C virus replication. J. Hepatol..

[28] Wu J., Meng Z., Jiang M., Zhang E., Trippler M., Broering R., Bucchi A., Krux F., Dittmer U., Yang D. (2010). Toll-like receptor-induced innate immune responses in non-parenchymal liver cells are cell type-specific. Immunology.

[29] Tu Z., Bozorgzadeh A., Pierce R.H., Kurtis J., Crispe I.N., Orloff M.S. (2008). TLR-dependent cross talk between human Kupffer cells and NK cells. J. Exp. Med..

[30] Visvanathan K., Skinner N.A., Thompson A.J., Riordan S.M., Sozzi V., Edwards R., Rodgers S., Kurtovic J., Chang J., Lewin S. (2007). Regulation of Toll-like receptor-2 expression in chronic hepatitis B by the precore protein. Hepatology.

[31] Knolle P.A., Gerken G. (2000). Local control of the immune response in the liver. Immunol. Rev..

[32] Su G.L., Goyert S.M., Fan M.H., Aminlari A., Gong K.Q., Klein R.D., Myc A., Alarcon W.H., Steinstraesser L., Remick D.G. (2002). Activation of human and mouse Kupffer cells by lipopolysaccharide is mediated by CD14. Am. J. Physiology.Gastrointest. Liver Physiol..

[33] Knolle P., Schlaak J., Uhrig A., Kempf P., Meyer zum Buschenfelde K.H., Gerken G. (1995). Human Kupffer cells secrete IL-10 in response to lipopolysaccharide (LPS) challenge. J. Hepatol..

[34] Karck U., Peters T., Decker K. (1988). The release of tumor necrosis factor from endotoxin-stimulated rat Kupffer cells is regulated by prostaglandin E2 and dexamethasone. J. Hepatol..

[35] Knolle P.A., Uhrig A., Protzer U., Trippler M., Duchmann R., Meyer zum Buschenfelde K.H., Gerken G. (1998). Interleukin-10 expression is autoregulated at the transcriptional level in human and murine Kupffer cells. Hepatology.

[36] Pestka S., Krause C.D., Walter M.R. (2004). Interferons, interferon-like cytokines, and their receptors. Immunol. Rev..

[37] de Weerd N.A., Samarajiwa S.A., Hertzog P.J. (2007). Type I interferon receptors: Biochemistry and biological functions. J. Biol. Chem..

[38] Kotenko S.V., Gallagher G., Baurin V.V., Lewis-Antes A., Shen M., Shah N.K., Langer J.A., Sheikh F., Dickensheets H., Donnelly R.P. (2003). IFN-lambdas mediate antiviral protection through a distinct class II cytokine receptor complex. Nat. Immunol..

[39] Prokunina-Olsson L., Muchmore B., Tang W., Pfeiffer R.M., Park H., Dickensheets H., Hergott D., Porter-Gill P., Mumy A., Kohaar I. (2013). A variant upstream of IFNL3 (IL28B) creating a new interferon gene IFNL4 is associated with impaired clearance of hepatitis C virus. Nat. Genet..

[40] Hamming O.J., Terczynska-Dyla E., Vieyres G., Dijkman R., Jorgensen S.E., Akhtar H., Siupka P., Pietschmann T., Thiel V., Hartmann R. (2013). Interferon lambda 4 signals via the IFNlambda receptor to regulate antiviral activity against HCV and coronaviruses. EMBO J..

[41] Robek M.D., Boyd B.S., Chisari F.V. (2005). Lambda interferon inhibits hepatitis B and C virus replication. J. Virol..

[42] Muir A.J., Shiffman M.L., Zaman A., Yoffe B., de la Torre A., Flamm S., Gordon S.C., Marotta P., Vierling J.M., Lopez-Talavera J.C. (2010). Phase 1b study of pegylated interferon lambda 1 with or without ribavirin in patients with chronic genotype 1 hepatitis C virus infection. Hepatology.

[43] Marcello T., Grakoui A., Barba-Spaeth G., Machlin E.S., Kotenko S.V., MacDonald M.R., Rice C.M. (2006). Interferons alpha and lambda inhibit hepatitis C virus replication with distinct signal transduction and gene regulation kinetics. Gastroenterology.

[44] Aka P.V., Kuniholm M.H., Pfeiffer R.M., Wang A.S., Tang W., Chen S., Astemborski J., Plankey M., Villacres M.C., Peters M.G. (2014). Association of the IFNL4-DeltaG Allele With Impaired Spontaneous Clearance of Hepatitis C Virus. J. Infect. Dis..

[45] Thomas E., Gonzalez V.D., Li Q., Modi A.A., Chen W., Noureddin M., Rotman Y., Liang T.J. (2012). HCV Infection Induces a Unique Hepatic Innate Immune Response Associated With Robust Production of Type III Interferons. Gastroenterology.

[46] Sutter K., Dickow J., Dittmer U. (2018). Interferon α subtypes in HIV infection. Cytokine Growth Factor Rev..

[47] Chen J., Li Y., Lai F., Wang Y., Sutter K., Dittmer U., Ye J., Zai W., Liu M., Shen F. (2021). Functional Comparison of Interferon-α Subtypes Reveals Potent Hepatitis B Virus Suppression by a Concerted Action of Interferon-α and Interferon-γ Signaling. Hepatology.

[48] Chen L., Borozan I., Sun J., Guindi M., Fischer S., Feld J., Anand N., Heathcote J., Edwards A.M., McGilvray I.D. (2010). Cell-type specific gene expression signature in liver underlies response to interferon therapy in chronic hepatitis C infection. Gastroenterology.

[49] Robinson M.W., Harmon C., O’Farrelly C. (2016). Liver immunology and its role in inflammation and homeostasis. Cell. Mol. Immunol..

[50] Heymann F., Peusquens J., Ludwig-Portugall I., Kohlhepp M., Ergen C., Niemietz P., Martin C., van Rooijen N., Ochando J.C., Randolph G.J. (2015). Liver inflammation abrogates immunological tolerance induced by Kupffer cells. Hepatology.

