# Supplementary material for: Antiviral Toll-like Receptor Signaling in Non-Parenchymal Liver Cells Is Restricted to TLR3
Source: Viruses. 2022 Jan 24;14(2):218. doi: 10.3390/v14020218 (PMC8874605; doi:10.3390/v14020218)

Supplementary Materials

Article

Antiviral Toll-like Receptor Signaling in Non-Parenchymal Liver Cells Is Restricted to TLR3

Melanie Werner [1,†,‡], Stefan Schefczyk [1,†,‡], Martin Trippler [1,‡], Juergen W. Treckmann [2], Hideo A. Baba [3], Guido Gerken [1,4,‡], Joerg F. Schlaak [1,5,‡] and Ruth Broering [1,*,‡]

[1] Department of Gastroenterology, Hepatology and Transplant Medicine, University Hospital of Essen, University Duisburg-Essen, Hufelandstr. 55, 45147 Essen, Germany; melanie.werner@uni-due.de (M.W.); stefan.schefczyk@uni-due.de (S.S.); Martin.Trippler@uk-essen.de (M.T.)
[2] Department of General-, Visceral- and Transplantation Surgery, University Hospital of Essen, University of Duisburg-Essen, Hufelandstr. 55, 45147 Essen, Germany; juergen-walter.treckmann@uk-essen.de (J.W.T.)
[3] Institute of Pathology, University Hospital of Essen, University of Duisburg-Essen, Hufelandstr. 55, 45147 Essen, Germany; hideo.baba@uk-essen.de (H.A.B.)
[4] Helios Hospital, Gastroenterology, Hepatology and Palliative Medicine, Robert-Koch-Straße 2, 42549 Velbert, Germany; guido.gerken@helios-gesundheit.de (G.G.)
[5] AMEOS Hospital, St. Clemens, Internal Medicine—Hepatology, Gastroenterology, Infectiology and Diabetology; Wilhelmstr. 34, 46145 Oberhausen, Germany; joerg.schlaak@ob.ameos.de (J.F.S.)
* Correspondence: ruth.broering@uni-due.de; Tel.: +49-201-723-6015
† Contributed equally.
‡ Former Dept. of Gastroenterology and Hepatology.

Content:

Supplementary Figures……………………… Page 2
Supplementary Tables………………………. Page 3
Original western blots………………………. Page 6

Supplementary Figures

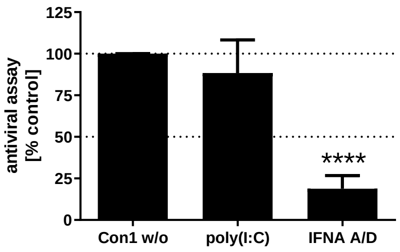

Figure S1. HCV replicon system con1 does not respond to poly(I:C). Subgenomic HCV replicon system con1 was treated with 50µg/ml poly(I:C) or 10IU/ml recombinant IFNA A/D. RNA was extracted after 72h and HCV replication was determined by qRT-PCR.

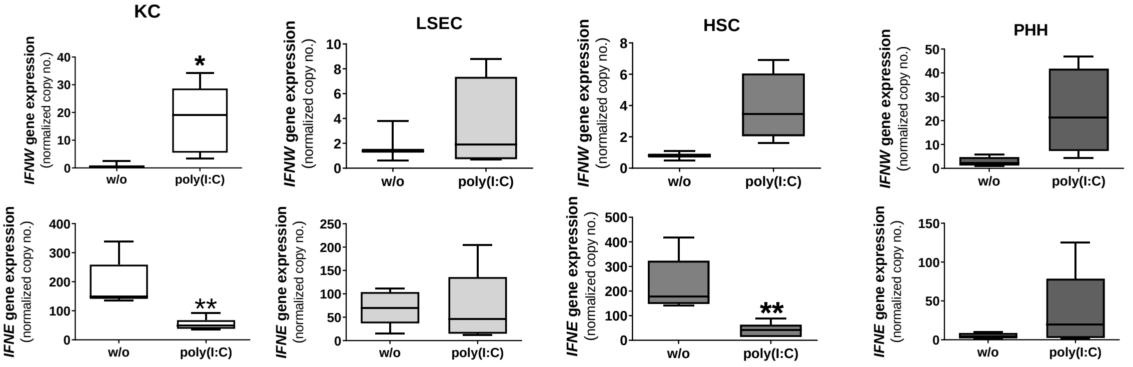

Figure S2. NPC show low expression of *IFNW* and *IFNE*. Gene expression of *IFNW* and *IFNE* was determined in NPC (n=3) treated with poly(I:C) for 6h. Data represent copy numbers as mean ± sd normalized to 100,000 copies of reference gene ACTB. Abbreviations: HSC, hepatic stellate cells; KC, Kupffer cells; LSEC, liver sinusoidal endothelial cells; nd, not detectable; sd, standard deviation; TLR, toll like receptor; w/o (without treatment)

Supplementary Tables

Table S1. Patients' characteristics

**KC**	**Control group (n=15)** **mean ± sem**	**HCV group (n=10)** **mean ± sem**
transplantation	3	9
resection	12	1
male	8	7
female	7	3
age	54.9±4.2	55.6±2.1
GOT [IU/ml]	214.0±57.7	241.4±119.6
GPT [IU/ml]	153.4±37.6	169.9±77.0
fibrosis stage	1.5±0.4	3.9±,0.1
**LSEC**	**Control group (n=15)** **mean ± sem**	**HCV group (n=10)** **mean ± sem**
transplantation	2	10
resection	13	0
male	9	7
female	6	3
age	56.7±4.6	55.0±1.1
GOT [IU/ml]	233.9±64.1	216.1±115.3
GPT [IU/ml]	141.0±40.6	163.8±77.5
fibrosis stage	1.7±0.4	3.7±0.3
**HSC**	**Control group (n=15)** **mean ± sem**	**HCV group (n=10)** **mean ± sem**
transplantation	2	10
resection	13	0
male	5	9
female	10	1
age	58.5±5.2	53.1±1.6
GOT [IU/ml]	277.3±88.6	222.3±114.1
GPT [IU/ml]	187.1±61.4	164.7±77.4
fibrosis stage	2.2±0.5	3.7±0.3

Data represent mean values±sem. Abbreviations: GOT, glutamate oxaloacetate transaminase; GPT, glutamate pyruvate transaminase; HCV, hepatitis c virus; HSC, hepatic stellate cells; KC, Kupffer cells; LSEC, liver sinusoidal endothelial cells; sem, standard error of mean.

Table S2. Primer sequences

**Gene**	Forward primer sequence	Reverse primer sequence	Species
ACTB	5′-TCCCTGGAGAAGAGCTACGA-3′	5′-AGCAATGTGTTGGCGTACAG-3′	Hs
HCV	5′-GCGGGTTGATCCAAGAAAGG-3′	5′-ATCACTCCCCTGTGAGGAACT-3′	Hs
MX1	5′-AGCCACTGGACTGACGACTT-3′	5′-GAGGGCTGAAAATCCCTTTC-3′	Hs
Ifit1	5′-CTGAAATGCCAAGTAGCAAGG-3′	5′-CCAAAGGCACAGACATAAGGA-3′	Mm
Gapdh	5′- AAATTCAACGGCACAGTCAA-3′	5′-TCTCCATGGTGGTGAAGACA-3′	Mm

Table S3. Primary and secondary antibodies

Protein	Abbreviation	Company
Interferon regulatory factor 3	IRF3	Abcam
Phospho-interferon regulatory factor 3	P-IRF3	Abcam
Nuclear factor kappa B	NF-κB	Cell Signaling
Phospho-nuclear factor kappa B	P-NF-κB	Cell Signaling
Mitogen-activated protein kinase (MAPK)	MAPK	Cell Signaling
Phospho-mitogen-activated protein kinase	P-MAPK	Cell Signaling
Protein 38	p38	Cell Signaling
Phospho-protein 38	P-p38	Cell Signaling
c-Jun-amino-terminal kinases	JNK	Cell Signaling
Phospho-c-Jun-amino-terminal kinases	P-JNK	Cell Signaling
Serine/threonine-protein kinase	AKT	Cell Signaling
Phospho-serine/threonine-protein kinas	P-AKT	Cell Signaling
Actin beta	ACTB	Sigma
Glyceraldehyde 3-phosphate dehydrogenase	GAPDH	GAPDH
Goat anti-rabbit HRP-conjugated	-	Cell Signaling
Goat anti-mouse HRP-conjugated	-	Thermo Scientific

Abbreviations: HRP, horseradish peroxidase

Table S4. TLR-induced gene expression of inflammatory cytokines

**KC**	*IL6*		*TNF*		*IL10*	
Stimulation	mean ± sd	p-value	mean ± sd	p-value	mean ± sd	p-value
w/o	9,862.3 ± 8,243.6		352.7 ± 164.2		148.9 ± 129.4	
Pam3CSK4	**35,275.5 ± 19,675.9**	**<0.001**	**2,509.5 ± 1,531.8**	**<0.001**	233.4 ± 121.6	0.071
HKLM	**47,645.5 ± 30,411.1**	**<0.001**	**4,297.4 ± 3,641.0**	**<0.001**	**467.4 ± 268.4**	**<0.001**
poly(I:C)	**27,112.3 ± 16,397.4**	**0.001**	**2,523.1 ± 1,394.3**	**<0.001**	151.8 ± 102.2	0.946
LPS	**49,002.0 ± 34,574.1**	**<0.001**	**2,833.9 ± 2,204.5**	**<0.001**	**346.7 ± 305.2**	**0.026**
flagellin	**87,368.8 ± 44,872.0**	**<0.001**	**10,402.4 ± 8,008.9**	**<0.001**	**544.9 ± 309.8**	**<0.001**
FSL-I	**39,569.6 ± 23,103.9**	**<0.001**	**3,599.6 ± 3,697.9**	**0.002**	**270.0 ± 177.0**	**0.037**
Gdq	**56,604.8 ± 38,768.2**	**<0.001**	**9,387.8 ± 5,379.6**	**<0.001**	**533.9 ± 345.9**	**<0.001**
ssRN40	**24,331.4 ± 12,833.9**	**0.001**	**3,358.6 ± 2,831.5**	**<0.001**	233.5 ± 209.5	0.190
ODN2216	12,280.9 ± 10,063.2	0.474	**695.6 ± 290.5**	**<0.001**	178.2 ± 139.0	0.552
**LSEC**	*IL6*		*TNF*		*IL10*	
Stimulation	mean ± sd	p-value	mean ± sd	p-value	mean ± sd	p-value
w/o	3,156.1 ± 2,819.5		244.0 ± 180.5		40.3 ± 35.6	
Pam3CSK4	4,428.9 ± 2,866.8	0.225	**512.4 ± 388.8**	**0.020**	52.0 ± 46.9	0.446
HKLM	4,160.7 ± 3,022.5	0.350	**522.4 ± 399.7**	**0.018**	46.6 ± 43.4	0.667
poly(I:C)	**13,250.3 ± 11,861.0**	**0.003**	**720.9 ± 496.9**	**0.001**	74.1 ± 55.0	0.065
LPS	**5,853.4 ± 3,562.4**	**0.025**	**710.6 ± 602.2**	**0.007**	58.3 ± 46.1	0.240
flagellin	**14,118.1 ± 19,502.0**	**0.039**	**896.8 ± 576.7**	**<0.001**	**94.1 ± 72.4**	**0.013**
FSL-I	**7,576.4 ± 3,537.6**	**<0.001**	**791.0 ± 500.8**	**<0.001**	64.7 ± 48.4	0.125
Gdq	4,165.8 ± 2,442.7	0.299	326.6 ± 326.1	0.395	44.0 ± 38.0	0.784
ssRN40	3,944.8 ± 2,480.3	0.419.	**493.5 ± 397.4**	**0.032**	34.7 ± 20.1	0.601
ODN2216	2,527.5 ± 1,969.0	0.482	216.0 ± 154.9	0.650	32.9 ± 24.4	0.521
**HSC**	*IL6*		*TNF*		*IL10*	
Stimulation	mean ± sd	p-value	mean ± sd	p-value	mean ± sd	p-value
w/o	11,396.2 ± 5,376.1		61.3 ± 60.0		12.2 ± 13.6	
Pam3CSK4	**38,148.5 ± 18,017.5**	**<0.001**	**316.9 ± 324.9**	**0.005**	25.2 ± 33.7	0.175
HKLM	**40,810.2 ± 21,262.3**	**<0.001**	**810.0 ± 1,059.6**	**0.011**	**43.5 ± 51.9**	**0.032**
poly(I:C)	**54,436.2 ± 23,117.5**	**<0.001**	**152.5 ± 156.5**	**0.046**	22.2 ± 22.5	0.198
LPS	**63,691.4 ± 27,297.2**	**<0.001**	**476.6 ± 634.8**	**0.017**	**41.1 ± 45.7**	**0.035**
flagellin	**132,965.1 ± 110,010.5**	**<0.001**	**899.9 ± 819.9**	**<0.001**	**57.0 ± 55.8**	**0.006**
FSL-I	**53,066.1 ± 52,041.3**	**0.004**	**319.3 ± 300.2**	**0.003**	24.4 ± 22.1	0.089
Gdq	**51,273.5 ± 50,818.6**	**0.005**	**489.7 ± 626.3**	**0.013**	24.0 ± 22.9	0.124
ssRN40	**31,228.8 ± 33,671.3**	**0.032**	**248.1 ± 338.9**	**0.044**	20.2 ± 16.7	0.199
ODN2216	15,212.0 ± 11,629.0	0.255	71.5 ± 53.1	0.631.	13.8 ± 13.2	0.774

Gene expression of inflammatory cytokines was determined in untreated NPC (w/o) or in cells treated with TLR ligands for 6h (n=15). Data represent copy numbers as mean ± sd normalized to 100,000 copies of reference gene *ACTB*. Abbreviations: FSL-I; synthetic lipoprotein (TLR2/6 ligand); Gdq, Gardiquimod (TLR7 ligand); HKLM, Heat Killed Listeria monocytogenes (TLR2 ligand); HSC, hepatic stellate cells; KC, Kupffer cells; LPS, lipopolysaccharide (TLR4 ligand); LSEC, liver sinusoidal endothelial cells; ODN2216, synthetic single-stranded DNA (TLR9 ligand); Pam3CSK4, synthetic triacylated lipopeptide (TLR1/2 ligand); poly(I:C), polyinosinic-polycytidylic acid (TLR3 ligand); sd, standard deviation; ssRNA40, single-stranded RNA oligonucleotide (TLR8 ligand); TLR, toll like receptor; w/o (without treatment)

Original western blots - Figure 1

KC

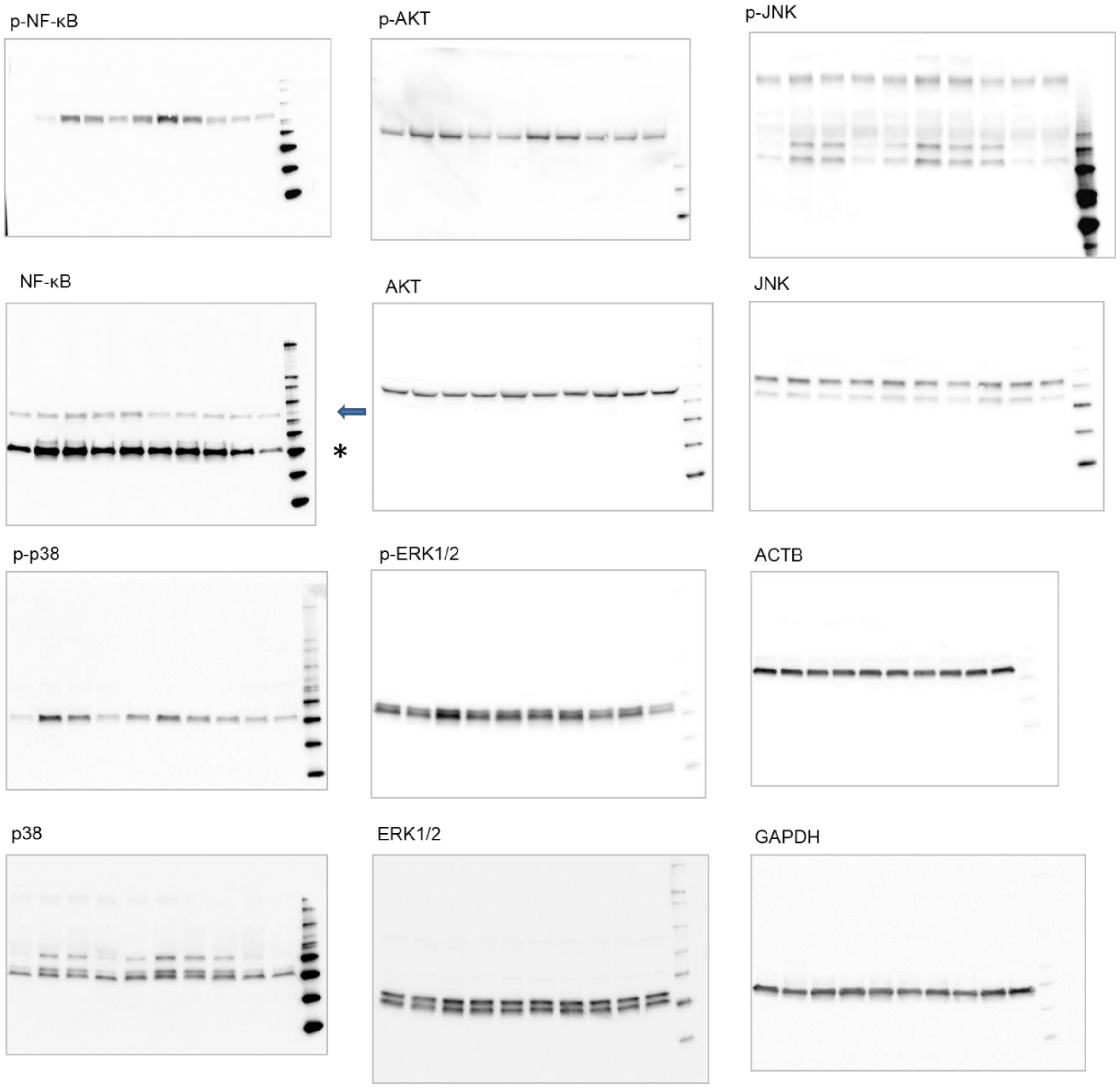

* Sequential use of blots for staining of proteins with different size.

⟸ band of interest

Original western blots - Figure 1

LSEC

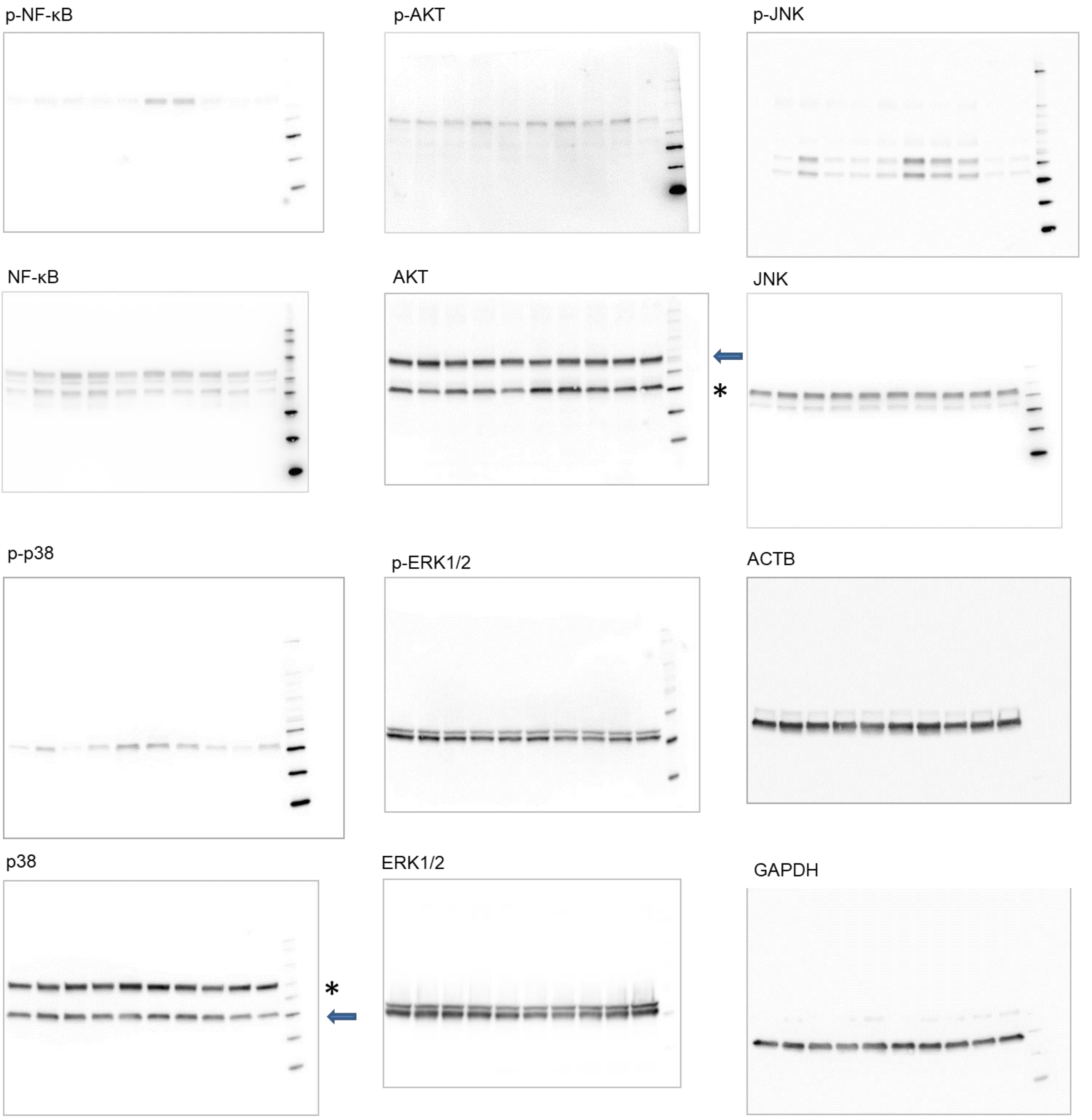

* Sequential use of blots for staining of proteins with different size.

band of interest

Original western blots - Figure 1

HSC

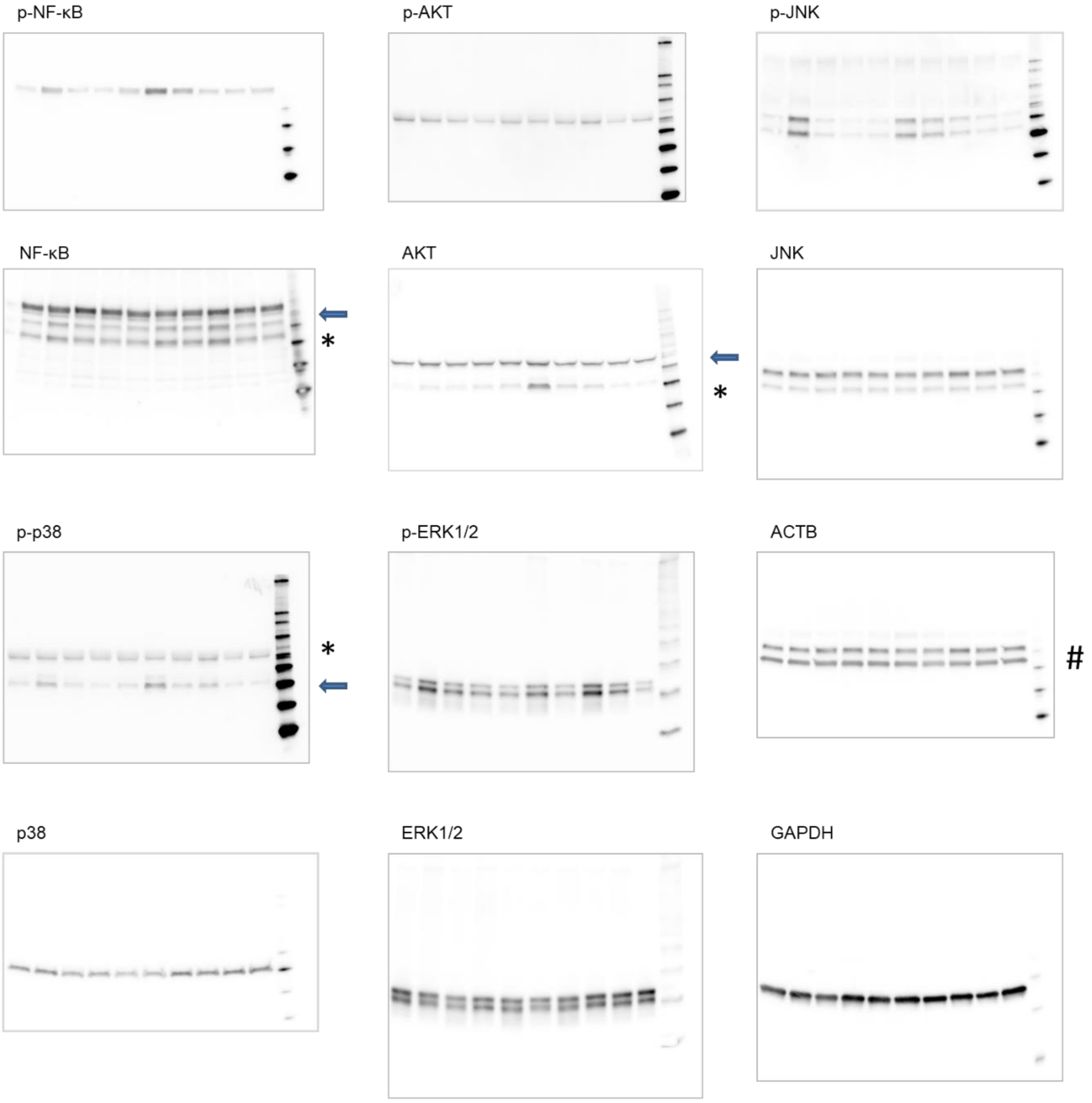

* Sequential use of blots for staining of proteins with different size.

⟸ band of interest

double band actin appeared in HSC

Original western blots - Figure 2

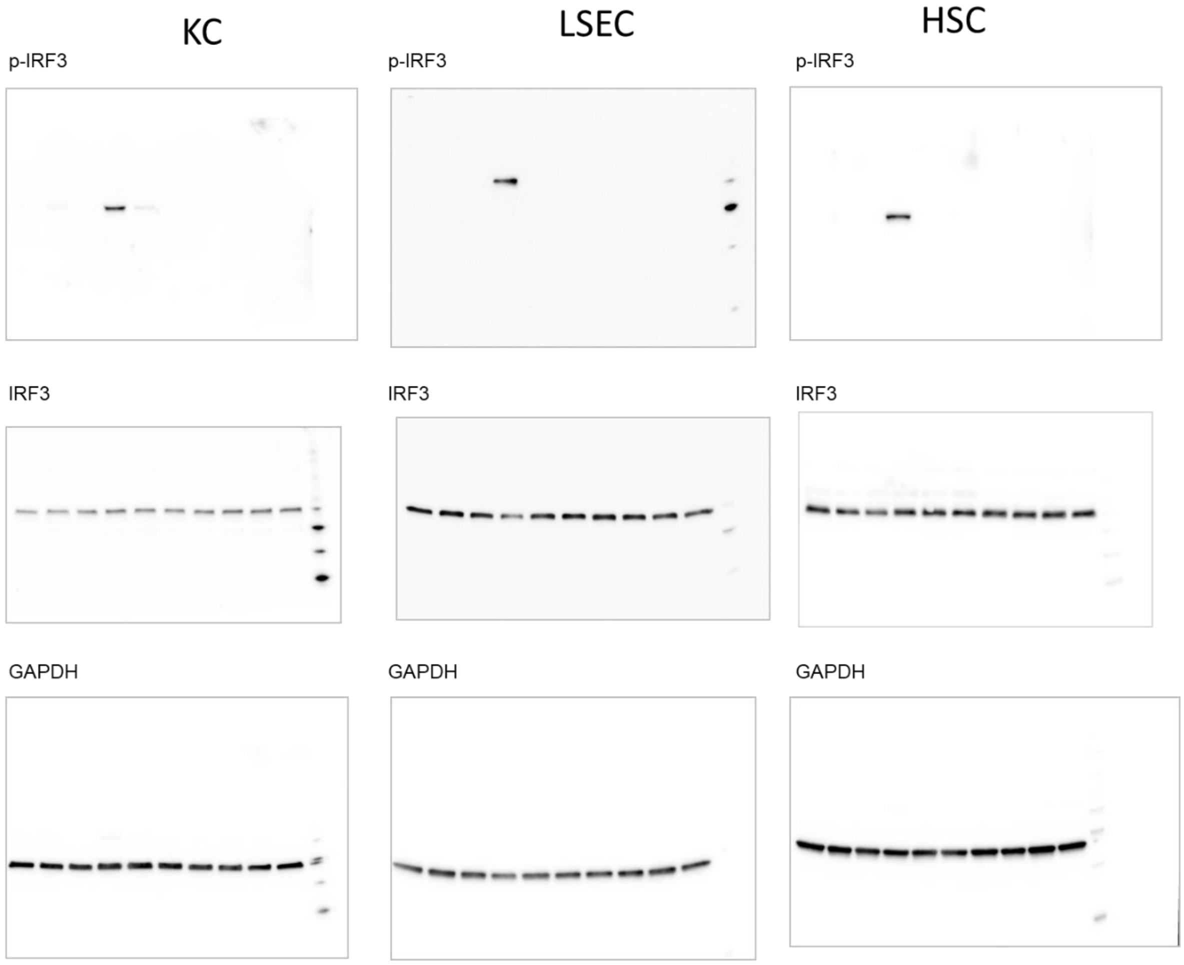

Supplement: Supplementary file 1 [file viruses-14-00218-s001.zip › viruses-1542452-supplementary.pdf]
